# Online vs. face-to-face interactive communication education using video materials among healthcare college students: a pilot non-randomized controlled study

**DOI:** 10.1186/s12909-024-05742-2

**Published:** 2024-07-10

**Authors:** Kanako Ichikura, Kazuhiro Watanabe, Rika Moriya, Hiroki Chiba, Akiomi Inoue, Yumi Arai, Akihito Shimazu, Yuko Fukase, Hirokuni Tagaya, Akizumi Tsutsumi

**Affiliations:** 1 Department of Health Science, Kitasato University School of Allied Health Sciences, 1-15-1 Kitazato, Minami-ku, Sagamihara, Kanagawa 252-0373 Japan; 2 Kitasato University Graduate School of Medical Sciences, 1-15-1 Kitazato, Minami-ku, Sagamihara, Kanagawa 252-0373 Japan; 3 Department of Medical Education, Kitasato University School of Medicine, 1-15-1 Kitazato, Minami-ku, Sagamihara, Kanagawa 252-0374 Japan; 4 Institutional Research Center, University of Occupational and Environmental Health, Japan, 1-1 Iseigaoka, Yahatanishi-ku, Kitakyushu, Fukuoka 807-8555 Japan; 5 Department of Public Health, Kitasato University School of Medicine, 1-15-1 Kitazato, Minami- ku, Sagamihara, Kanagawa 252-0374 Japan; 6 Department of Patient Safety and Hospital Administration, Kitasato University Hospital, 1-15-1 Kitazato, Minami-ku, Sagamihara, Kanagawa 252-0375 Japan; 7 Faculty of Policy Management, Keio University, 5322, Endo, Fujisawa, Kanagawa 252-0882 Japan

**Keywords:** Healthcare communication, Online, Face-to-face, Communication exercise, Communication education

## Abstract

**Background:**

This study aimed to examine whether online interactive communication education using video materials was as effective as face-to-face education among healthcare college students.

**Methods:**

The participants were healthcare college students who were enrolled in study programs to obtain national medical licenses. They participated in lectures and exercises on healthcare communication, both online (*n* = 139) and face-to-face (*n* = 132). Listening skills, understanding, and confidence in healthcare communication were assessed using a self-assessed tool.

**Results:**

From the two-way ANOVA result, the interaction effects between group (online, face-to-face) and time (Time 1, Time 2, Time 3) were not statistically significant. The main effect of time increased significantly from Time1 to Time 3 on understanding of communication with patients (Hedges’g = 0.51, 95%CI 0.27–0.75), confidence in communication with patients (g = 0.40, 95%CI 0.16–0.64), and confidence in clinical practice (g = 0.49, 95%CI 0.25, 0.73), while the score of listening skills had no significant change (Hedges’g = 0.09, 95%CI − 0.03 to 0.45).

**Conclusions:**

The results show that online communication education with video materials and active exercises is as effective in improving students’ confidence as face-to-face. It will be necessary to modify the content of this educational program to improve skills as well as confidence in communication.

**Trial registration:**

Not Applicable.

**Supplementary Information:**

The online version contains supplementary material available at 10.1186/s12909-024-05742-2.

## Background

The term healthcare communication refers to interactive conversations between patients and healthcare professionals to understand symptoms and deliver professional knowledge or information. Good communication contributes to patient–healthcare professional trust building and increased treatment understanding, satisfaction, adherence, and quality of life among patients [1–3]. By contrast, discrepancies in healthcare communication can lead to decreased satisfaction and burnout among healthcare professionals [4, 5]. These descriptions showcase how crucial communication skills are among healthcare professionals as they demonstrate the professional’s expertise.

Communication skills can be improved through practical training programs [6]. Moreover, in clinical practice, patients are more likely to feel satisfied when they interact with healthcare professionals who have been trained in communication skills and, in turn, show empathy toward them (e.g., make eye contact, use gestures, and pay attention to the other’s nonverbal cues) [7]. There are studies showcasing that practical training is effective in improving communication skills not only among healthcare professionals but also among students who aspire to become healthcare professionals [8–10]. Accordingly, healthcare communication classes have recently been incorporated into the pre-graduate education curriculum in Japan.

In healthcare communication education, it is important to provide students with practical and theoretical knowledge of the related skills. Role-playing activities have long been a common method used in communication classes for developing practical knowledge. In healthcare settings, role-play refers to exercises wherein students play the roles of healthcare professionals and patients in the context of given scenarios, and these activities are believed to improve their self-understanding and skills as healthcare professionals [11–13]. In these activities, the process of becoming aware of various and divergent perspectives (i.e., the patient, healthcare professional, and observer) is important [10]. Therefore, it is key for students to have opportunities to think about communication between healthcare professionals and patients from multiple perspectives, even if not through role-playing.

In recent years, online education has become popular worldwide owing to the development of online learning content and conference systems. The COVID-19 pandemic also provided new and unprecedented challenges for face-to-face education, scaffolding the increase in demand for online education [14]. Moreover, several studies have shown that online classes contribute to the improvement of healthcare students’ knowledge and skills [15] and the contributions of online healthcare communication education to healthcare professionals’ knowledge and confidence [16]. Concomitantly, a study conducted with medical students described that unidirectional education using digital content might not lead to improved communication skills and knowledge [17]. There is also evidence depicting that non-practical education, such as small group discussions, is not as effective as role-play for communication training [18]. These descriptions imply that online healthcare communication education may require practical and interactive training to increase the scope of perspectives regarding the communication skills of healthcare professionals and students.

As aforementioned, the process of confirming the meaning of basic communication skills learned in theory through practical application is important for deepening the learning process of healthcare professionals. In a prior study, the authors of this paper developed communication education content that provides practical training without role-playing or face-to-face lectures [19]. This educational material encompasses a counseling video wherein there are images of the professional and patient talking, but the healthcare professional’s voice is silent in response to the patient’s complaint. This is intended to train students to think about how they should talk to patients from the standpoint of a healthcare professional. Since previous studies have shown that combining e-learning and role-play learning is effective in communication education [20], in a past study of ours [19], we conducted exercises using the material we developed in an online lecture after students had engaged in a prior e-learning activity [21]. The findings of our past study showed a certain level of effectiveness for the online lecture on communication skills and knowledge [19]. However, it remains that few studies have examined the effectiveness of online healthcare communication education for healthcare college students compared to face-to-face education. Furthermore, online healthcare communication education has challenges in practicality and interactivity, as it is difficult to ensure students’ active participation and engagement in communication exercises [17, 18]. This study aimed to compare the effectiveness of interactive online healthcare communication education provided by video materials with that of face-to-face education on communication skills and knowledge.

## Methods

### Design and participants

This pilot non-randomized controlled study compared the effectiveness of online healthcare communication education conducted in July 2020 and face-to-face classes conducted in July 2021. In 2020, 139 undergraduate students who had taken courses to obtain medical qualifications and 10 graduate students who had taken courses to obtain clinical psychologists licenses participated in this online healthcare communication lecture at *** University, ** (country). In 2021, 145 similar undergraduate students and 10 graduate students participated in the face-to-face lecture. The instructor is a university lecturer who specializes in clinical psychology, is a licensed clinical psychologist, and has five years of experience in health communication exercises. Students participated in a total of 15 “Developmental Psychology” classes, one of which featured a lecture on health communication. The classes were established as a course of study for being qualified as healthcare professionals, and students aiming to become certified clinical psychologists, physical therapists, occupational therapists, speech-language pathologists, and optometrists participated in these lectures. Prior to the lecture in both the online and face-to-face methods, participants engaged in e-learning pre-training homework. Then, they participated a 90-minute lecture with a counseling video-based communication exercise. At three time points during the lectures, a self-administered, anonymous, and voluntary questionnaire using Google Forms was administered, as follows: Time 1 before the e-learning pre-training homework (one week before the lecture), Time 2 after the e-learning pre-training (immediately before the lecture), and Time 3 after the role-play exercise (after the lecture). Sex, age, and aimed licenses were also asked to be answered in the self-administered form.

### Interventions

#### Face-to-face healthcare communication lectures

The contents of the communication training lectures are shown in Table 1. Before the lecture, students were asked to watch the e-learning materials (30 min) that we had developed as homework [19, 22]. Through these materials, they learned about basic communication skills, such as reflection techniques and healthcare communication with patients experiencing a psychological crisis (Appendix 1).

**Table 1:** The contents of the healthcare communication lectures

	Topic	Teachers’ action in the lecture	
		Face-to-face	Online
**Questionnaire (Time1: Pre training using e-learning)**			
E-learning pre-training homework (30 min)			
Basic communication (Appendix 1)	•Basic listening skills (e.g., make eye contact, speak in a low tone, observe the patient closely) •Reflection technique to impress patients (e.g., nodding and back-channeling, reflection of feeling, reflection of meaning, reflection of content)	Ask students to watch the e-learning materials as their pre-training homework.	Ask students to watch the e-learning materials as their pre-training homework.
Healthcare communication	•Communicating with patients experiencing a psychological crisis (e.g., patients who are frustrated, cannot stop talking, or have been given bad news)	Ask students to watch the e-learning materials as their pre-training homework.	Ask students to watch the e-learning materials as their pre-training homework.
**Questionnaire (Time 2: Post e-learning)**			
Lecture (90 min)			
Review of e-learning pre-training homework (Appendix 2)	• Confirmation quiz of listening skills Read the text of the conversation between the healthcare provider and the patient, and answer on the sheet which reflection technique was used.	1. Ask students to provide answers about the reflection technique used by the healthcare provider in each statement. 2. **Request some of the students present in the lecture to tell the answers they wrote down.** 3. **Answer correctly or incorrectly.**	1. Ask students to provide answers about the reflection technique used by the healthcare provider in each statement. 2. **Give the right answer.**
Healthcare communication skills training using a counseling video (Appendix 3)	• Practical listening skill exercise Watch the conversation video between the healthcare provider and the patient, think of the blank lines of healthcare professionals, and fill them in.	1. Ask students to answer how they would talk to the patients in the conversation video. 2. **Request some of the students present in the lecture to tell the answers they wrote down**. 3. Provide positive feedback on students’ responses and support their attitudes of developing their own answer. 4. **Reflect on some desirable responses**, share them with the entire class, and communicate that there is no right or wrong response to a patient.	1. Ask students to answer how they would talk to the patients in the conversation video. 2. Ask students to **post the responses** they wrote down **in the chat section of the online conference system.** 3. Provide positive feedback on students’ responses and support their attitudes of developing their own answer. 4. **Pick out a few desirable responses posted in the chat**, share them with the entire class, and communicate that there is no right or wrong response to a patient.
**Questionnaire (Time 3: Post lecture)**			

Students then reviewed the e-learning homework and participated in healthcare communication exercises during the lecture (90 min). First, students conducted a confirmation quiz about the homework, which asked students to read the text of the conversation between the healthcare provider and the patient and to answer on the sheet which reflection technique (nodding and back-channeling, reflection of feeling, reflection of meaning, reflection of content) was used by healthcare professionals during the communication (Appendix 2).

Second, students participated in a practical listening skills exercise using conversation video materials we developed that included a conversation between a healthcare provider and a patient who complained of poor health and insomnia (Appendix 3) [19]. They were asked to watch the video, think of the blank lines left by healthcare professionals during communication, and provide relevant responses. At the end of the exercise, the lecturer questioned the students about their answers and requested that they tell the lecturer the answers they had written down. The lecturer shared the responses with the entire classroom while providing positive feedback on the responses.

#### Online healthcare communication lectures

The basic contents of the online healthcare communication lectures were similar to those of the face-to-face healthcare communication lectures (Table 2). However, the lecturer here asked students to post the responses they wrote down in the chat section of the online conference system (i.e., Zoom) and selected a few desirable responses provided in the chat.

**Table 2:** Characteristics of participants at the baseline

	Face-to-face lecture *N* = 132	Online lecture *N* = 139	
	n (%)	n (%)	*χ* ^2^
Sex			0.18
Men	44 (33.3)	43 (30.9)	
Women	88 (66.7)	96 (69.1)	
Grade			22.01^*^
First-year university students	0(0.0)	0 (0.0)	
Second-year university students	96 (72.7)	126 (90.6)	
Third-year university students	27 (20.5)	4 (2.9)	
Fourth-year university students	0 (0.0)	1 (0.7)	
First-year graduate students	9 (6.8)	8 (5.8)	
Second-year graduate students	0 (0.0)	0 (0.0)	
Aimed licenses (multiple responses)			24.10^*^
Clinical psychologist	20 (15.2)	11 (7.9)	
Physical therapist	35 (26.5)	44 (31.7)	
Occupational therapist	6 (4.5)	18 (12.9)	
Speech therapist	25 18.9)	34 (24.5)	
Orthoptist	30 (22.7)	31 (22.3)	
others	16 (12.1)	1 (0.7)	

*p* < .05^*^

### Instruments

#### Demographic characteristics

Participants’ sociodemographic data, namely sex, age, grade, and target licenses, were collected on the face sheet of the questionnaire (Table 2).

#### Listening skills

Listening skills were evaluated as the primary outcome using the 17-item self-rated scale for listening and speaking skills [23]. It is responded to on a 5-point Likert-type scale (1, do not agree; 5, completely agree) ranging from 18 to 85. This scale has been standardized by a previous study (α = 0.92) and is widely used as a measure of counselor’s attitude [23].

#### Understanding and confidence in healthcare communication

No researcher thus far has developed a standardized self-assessed measurement tool for healthcare communication understanding and confidence. We used a visual analog scale to check students’ understanding of lectures and confidence in healthcare communication for educational purposes, using three items developed by the lecturer (i.e., “understanding of communication with patients,” “confidence in communicating with patients,” and “confidence in clinical practice”) on a 0–100% scale. The results of this questionnaire were used as the outcome in this study.

### Sample size

The results of this study were based on an analysis of data collected from all students who participated in the practical healthcare communication exercises, and no sample size calculations were performed a priori. Therefore, we conducted a post hoc power analysis to detect the difference in changes in healthcare communication skills between two groups (i.e., online and face-to-face lectures) using G*Power version 3.1 [24].

### Statistical analysis

In this study, a two-way analysis of variance (group × time) was conducted to compare the effectiveness of the online and face-to-face lectures. The main effects of time and the interaction effect were referred to as the effectiveness of each and the differences between the two groups, respectively. Effect sizes (Hedges’g) and 95% confidence intervals (CIs) were calculated and examined for the main effects of time, and the significance level was set at 5%. All data were analyzed using SPSS Statistics for Windows version 28.

## Results

Of all the students, 139 who attended the online lectures in 2020 and 132 who attended the face-to-face lectures in 2021 responded to the questionnaire. They were all students with no prior practical training experience, and this was their first time participating in a health communication exercise lecture. The participants’ demographic characteristics are presented in Table 2. In both groups, the sex ratio was 1:2, and most participants were second- and third-year university students. Table 2 shows the frequency of the healthcare national licenses that the participants are aiming to acquire. In addition, the Cronbach’s alpha coefficient for the listening skills scale was 0.958.Of all the students, 139 who attended the online lectures in 2020 and 132 who attended the face-to-face lectures in 2021 responded to the questionnaire. They were all students with no prior practical training experience, and this was their first time participating in a health communication exercise lecture. The participants’ demographic characteristics are presented in Table 2. In both groups, the sex ratio was 1:2, and most participants were second- and third-year university students. Table 2 shows the frequency of the healthcare national licenses that the participants are aiming to acquire. In addition, the Cronbach’s alpha coefficient for the listening skills scale was 0.958.

Second, we used a two-way analysis of variance to compare the effects of online and face-to-face healthcare communication education (Table 3). The results confirmed that the interaction effects between group (online, face-to-face) and time (Time 1, Time 2, Time 3) were not statistically significant for understanding of communication with patients (*F* (2, 1) = 0.42, *p* = .66), confidence in communication with patients (*F* (2, 1) = 0.13, *p* = .88), confidence in clinical practice (*F* (2, 1) = 0.01, *p* = .99), or listening skill (*F* (2, 1) = 0.15, *p* = .86). For the main effects of each lecture, understanding of communication with patients, confidence in communication with patients, and confidence in clinical practice increased significantly from Time 1 to Time 3 in the online and face-to-face education groups (Hedges’g = 0.51, 95%CI 0.27–0.75, *p* = .00; g = 0.40, 95%CI 0.16–0.64, *p* = .00; g = 0.49, 95%CI 0.25, 0.73, *p* = .00). There was no significant increase in listening skill from Time 1 to Time 3 (Hedges’g = 0.09, 95%CI − 0.03 to 0.45, *p* = .09).

**Table 3:** Effect of medical communication exercises between face-to-face and online lecture

	Face-to-face						Online								
		M ± SD	Hedges’ g	95% CI	*p*			M ± SD	Hedges’ g	95% CI	*p*		*F* (Time*group)	*η* ^*2*^	*p*
Understanding of patient communication													0.42	0.00115	0.66
	**Time1** (***n*** = **132**)	57.59 ± 22.89	-	-	-		**Time1** (***n*** = **139**)	60.51 ± 17.44	-	-	-				
	**Time2** (***n*** = **112**)	60.80 ± 20.55	0.15	− 0.11 to 0.40	0.26		**Time2** (***n*** = **136**)	66.60 ± 16.20	0.36	0.12 to 0.60	0.00^*^				
	**Time3** (***n*** = **83**)	64.01 ± 17.26	0.31	0.03 to 0.58	0.02^*^		**Time3** (***n*** = **132**)	69.05 ± 15.78	0.51	0.27 to 0.75	0.00^*^				
Confidence in patient communication													0.13	0.08002	0.88
	**Time1** (***n*** = **132**)	49.77 ± 23.29	-	-	-		**Time1** (***n*** = **139**)	52.79 ± 18.79	-	-	-				
	**Time2** (***n*** = **112**)	51.47 ± 22.15	0.08	− 0.18 to 0.33	0.56		**Time2** (***n*** = **136**)	56.27 ± 18.31	0.19	− 0.05 to 0.42	0.12				
	**Time3** (***n*** = **83**)	55.94 ± 21.00	0.27	0.00 to 0.55	0.05		**Time3** (***n*** = **132**)	60.09 ± 17.48	0.40	0.16 to. 64	0.00^*^				
Confidence in clinical practice													0.01	0.00003	0.99
	**Time1** (***n*** = **132**)	42.33 ± 24.20	-	-	-		**Time1** (***n*** = **139**)	47.10 ± 20.15	-	-	-				
	**Time2** (***n*** = **112**)	47.35 ± 23.00	0.21	− 0.04 to 0.46	0.10		**Time2** (***n*** = **136**)	52.50 ± 18.90	0.28	0.04 to 0.51	0.02^*^				
	**Time3** (***n*** = **83**)	51.43 ± 20.64	0.40	0.12 to 0.67	0.00^*^		**Time3** (***n*** = **132**)	56.71 ± 18.80	0.49	0.25 to 0.73	0.00^*^				
Listening skill													0.15	0.00042	0.86
	**Time1** (***n*** = **132**)	53.20 ± 9.99	-	-	-		**Time1** (***n*** = **139**)	49.35 ± 11.30	-	-	-				
	**Time2** (***n*** = **112**)	53.20 ± 10.77	0.00	− 0.25 to 0.25	0.99		**Time2** (***n*** = **136**)	50.40 ± 11.46	0.09	− 0.14 to 0.33	0.44				
	**Time3** (***n*** = **83**)	55.48 ± 8.66	0.24	− 0.02 to 0.51	0.09		**Time3** (***n*** = **132**)	51.92 ± 13.39	0.21	− 0.03 to 0.45	0.09				

*p* < .05^*^; M = Mean; SD = Standard deviation; MD = Mean Difference

Finally, using a total sample size of 734 (all data acquired at 3 points among two groups) and each effect size (Partial Eta Squared Value) of the interactions obtained from our study, a post hoc power analysis was conducted under a significance level of α of 0.05, 2 degrees of freedom, and the number of groups of 6. The power of each outcome was estimated at 0.15, 0.08, 0.05, and 0.09.

## Discussion and conclusion

### Discussion

Our findings suggest that practical and interactive online healthcare communication education using counseling videos with blank lines is as effective as face-to-face communication education among healthcare college students. Since the spread of COVID-19, communication exercises utilizing group work with online conferencing systems have been conducted [25–27], but it is difficult to use such methods in a large-group lecture such as this study. Therefore, we believe online healthcare communication education with the video materials developed in this study provide a noteworthy example in the context of university education.

Another important implication is that the online lectures were as effective as face-to-face lectures in improving students’ comprehension and confidence in healthcare communication and clinical internships for healthcare college students. Thus, online communication skills training was found to be more effective in improving students’ self-efficacy than expected in previous studies [26, 27]. Nevertheless, the power for the difference between the effects of the two groups was low in this study, implying that a larger sample size may have produced a more significant difference. However, this difference was small compared to the improvement in each outcome caused by the lectures, and it was unlikely to be a practically meaningful difference, given that the main effect of time is well recognized in this study. Therefore, we can infer that there was no difference in effectiveness between the online and face-to-face groups.

The greatest strength of this study is its approach to interactivity [17] and practicality [18], which are challenges in online healthcare communication education. In addition, there are three possible reasons for the effectiveness of the online healthcare communication program used in this study. First, the e-learning pre-training homework emphasized the importance of active listening, empathy, and clear communication, providing students with strategies and techniques to improve their interactions with patients. The e-learning materials used in this study focused on micro-counseling skills, a basic model of counseling, and provided clear examples of good and bad practices [21]. In recent years and with the influence of the COVID-19 pandemic, e-learning education has rapidly developed and been accepted by students [28]. In healthcare communication lectures for healthcare college students, e-learning alone is expected to be sufficient to improve their perceived communication skills [29] and to be an effective pre-learning tool for role-play application in communication education for clinical psychology students [20]. Therefore, we believe that the pre-training using an e-learning approach contributed significantly to improving students’ confidence in healthcare communication.

Second, the healthcare provider–patient conversation video with blank lines was a practical exercise implemented in the lectures and may have contributed to improving healthcare college students’ confidence in communicating with patients. This video was developed by the authors of this paper in prior research and is expected to have effects similar to those of role-playing [19]. No previous studies have used similar video exercises for healthcare communication education, and thus the effectiveness of these exercises should be independently verified in the future.

Third, only online methodologies allow for students to share their opinions using a chat function, and this may have enhanced the understanding of communication skills of students who participated in the lectures. Students have been reported to have a preference for text interactions, which can be enabled by chat functions that allow students to ask questions during online lectures, and thus this can be used as a new educational strategy [30]. In particular, Japanese students are not very keen on speaking up and discussing in group lectures, and the chat may have made it easier for these students to express their opinions [31].

However, healthcare communication lectures did not improve students’ actual communication skills as much as they did improve their confidence in communication. Previous studies have suggested that active and practical strategies are essential for improving communication skills, even among healthcare professionals [32]. Moreover, it may be difficult for students to acquire communication skills through an educational program alone, seeing that even communication education that utilizes simulated-patient and role-play activities reportedly has a limited effect on healthcare students’ acquisition of communication skills [33]. Therefore, further consideration should be given to the content of online communication exercises. Additionally, comparative studies with different participants and settings are required to develop educational programs that consider factors such as learning style, age group, and area of expertise.

This study has five limitations. First, this study is merely a quasi-experiment and not a randomized controlled trial. However, it is interesting that the results show that online interactive healthcare communication education, which was conducted under necessity during the COVID-19 pandemic, was as effective as face-to-face education. Second, the possible differences between the two groups could not be adequately established a priori as hypotheses. If a very small effect size should have been detected, the sample size was insufficient and statistical power was low. Third, it compared data from a cross-sectional survey at three time points and did not compare data longitudinally at the three time points. However, the data were collected during educational lectures, and the samples at the three time points were generally homogeneous. Fourth, this was a single-center study, limiting the generalizability of the programs presented and the study findings. Finally, the outcomes were self-assessed and did not indicate objective changes in the comprehension and skills of students regarding healthcare communication.

### Conclusions

This study showed that e-learning pre-training activities and the implementation of counseling videos with blank lines improved healthcare college students’ confidence in healthcare communication in online lectures as well as in face-to-face lectures. This study shows that e-learning, pre-learning activities and counseling videos with blank lines improve college students’ confidence in healthcare communication in online lectures as well as in face-to-face lectures. In so doing, this research configures an impressive first step toward the development of online education in the field of healthcare communication. In the future, the content of related online educational programs should be further refined to secure the development of healthcare professionals with good communication skills.

### Practical implications

An important implication of our study is that the effectiveness of online communication exercises is not inferior to face-to-face, but rather has additional potential. Communication exercises in pre-graduate education should be used more widely online in the future.

### Electronic supplementary material

Below is the link to the electronic supplementary material.

Supplementary Material 1

## Data Availability

The datasets generated and/or analyzed during the current study are not publicly available as this permission was not obtained in the informed consent form, however, data are available from the corresponding author on reasonable request.

## Abbreviations

Cis: Confidence intervals

## References

[1] Kim SS, Kaplowitz S, Johnston MV (2004). The effects of physician empathy on patient satisfaction and compliance. Eval Health Prof.

[2] Neumann M, Wirtz M, Bollschweiler E, Mercer SW, Warm M, Wolf J (2007). Determinants and patient-reported long-term outcomes of physician empathy in oncology: a structural equation modelling approach. Patient Educ Couns Patient ed.

[3] Takayama T, Yamazaki Y, Katsumata N (2001). Relationship between outpatients’ perceptions of physicians’ communication styles and patients’ anxiety levels in a Japanese oncology setting. Soc Sci Med.

[4] Ramirez AJ, Graham J, Richards MA, Cull A, Gregory WM, Leaning MS (1995). Burnout and psychiatric disorder among cancer clinicians. Br J Cancer.

[5] Ramirez AJ, Graham J, Richards MA, Cull A, Gregory WM (1996). Mental health of hospital consultants: the effects of stress and satisfaction at work. Lancet.

[6] Wolstencroft J, Robinson L, Srinivasan R, Kerry E, Mandy W, Skuse D (2018). A systematic review of group social skills interventions, and meta-analysis of outcomes, for children with high functioning ASD. J Autism Dev Disord.

[7] Patel S, Pelletier-Bui A, Smith S, Roberts MB, Kilgannon H, Trzeciak S (2019). Curricula for empathy and compassion training in medical education: a systematic review. PLoS ONE.

[8] D’souza PC, Rasquinha SL, D’souza TL, Jain A, Kulkarni V, Pai K (2020). Effect of a single-session communication skills training on empathy in medical students. Acad Psychiatry.

[9] Son D, Shimizu I, Ishikawa H, Aomatsu M, Leppink J (2018). Communication skills training and the conceptual structure of empathy among medical students. Perspect Med Educ.

[10] Vogel D, Meyer M, Harendza S (2018). Verbal and non-verbal communication skills including empathy during history taking of undergraduate medical students. BMC Med Educ.

[11] Bagacean C, Cousin I, Ubertini AH, El Yacoubi El Idrissi M, Bordron A, Mercadie L (2020). Simulated patient and role play methodologies for communication skills and empathy training of undergraduate medical students. BMC Med Educ.

[12] Gelis A, Cervello S, Rey R, Llorca G, Lambert P, Franck N (2020). Peer role-play for training communication skills in medical students: a systematic review. Simul Healthc.

[13] Lavanya SH, Kalpana L, Veena RM, Bharath Kumar VD (2016). Role-play as an educational tool in medication communication skills: students’ perspectives. Indian J Pharmacol.

[14] Mao S, Guo L, Li P, Shen K, Jiang M, Liu Y (2023). New era of medical education: asynchronous and synchronous online teaching during and after COVID-19. Adv Physiol Educ.

[15] Tang B, Coret A, Qureshi A, Barron H, Ayala AP, Law M (2018). Online lectures in undergraduate medical education: scoping review. JMIR Med Educ.

[16] Berg MN, Ngune I, Schofield P, Grech L, Juraskova I, Strasser M (2021). Effectiveness of online communication skills training for cancer and palliative care healthcare professionals: a systematic review. Psycho-oncology.

[17] Kyaw BM, Posadzki P, Paddock S, Car J, Campbell J, Tudor Car L (2019). Effectiveness of digital education on communication skills among medical students: systematic review and meta-analysis by the digital health education collaboration. J Med Internet Res.

[18] Krishnan DG, Keloth AV, Ahmad S, Pg M (2023). Role play versus small group discussion in teaching prescription communication skills: a comparative study on students of phase two of the bachelor of medicine and bachelor of surgery (MBBS) course. J Adv Med Educ Prof.

[19] Ichikura K, Moriya R, Chiba H, Inoue A, Watanabe K, Arai Y (2022). Feasibility and effectiveness of online lecture-based medical communication skills training using a counseling video [Japanese]. J Jpn Assoc Health Commun.

[20] Goosse M, Van der Kreusch F, Willems S (2023). Impact of e-learning and role play-based training on psychology students’ communication skills: a feasibility study. J Commun Healthc.

[21] Chiba H, Inoue A, Moriya R, Shimazu A, Ichikura K, Tsutsumi A (2021). Development of a video on learning active listening to facilitate role playing exercise for medical students [Japanese]. J Jpn Assoc Simul Med Educ.

[22] Inoue A, Tsutsumi A, Moriya R, Chiba H, Shimazu A, Kanako I (2020). Practice of behavioral science/behavioral medicine education at Kitasato University School of Medicine [Japanese]. Jpn J Behav Med.

[23] Ishii K, Arai K (2017). Construction of scales for listening and speaking skills and their relation to external and internal adaptation [Japanese]. Bull Clin Psychol Tokyo Seitoku Univ.

[24] Faul F, Erdfelder E, Lang AG, Buchner A (2007). G*Power 3: a flexible statistical power analysis program for the social, behavioral, and biomedical sciences. Behav Res Methods.

[25] Suryadinata N, Eka NGA, Manik MJ, Puspitasari V, Marlina M, Houghty GS (2024). Effectiveness of online interprofessional education-communication course during the COVID-19 pandemic. Heliyon.

[26] Can Ş, Durgun H, Dalcalı BK (2022). Effect of online communication skills training on effective communication and self-efficacy and self-regulated learning skills of nursing students: a randomized controlled study. Nurse Educ Pract.

[27] Yang J, Kim S (2022). An online communication skills training program for nursing students: a quasi-experimental study. PLoS ONE.

[28] Naciri A, Radid M, Kharbach A, Chemsi G (2021). E-learning in health professions education during the COVID-19 pandemic: a systematic review. J Educ Eval Health Prof.

[29] Soundy A, Hemmings L, Gardiner L, Rosewilliam S, Heneghan NR, Cronin K (2021). E-learning communication skills training for physiotherapy students: a two phased sequential mixed methods study. Patient Educ Couns.

[30] Vu P, Fadde PJ (2013). When to talk, when to chat: student interactions in live virtual classrooms. J Interact Online Learn.

[31] Kabashima M. Can online lectures using chat and reactions be a new form of independent learning? Practical report on Online lectures at Kyoto Sangyo University, 13. Forum High Educ Res. 2023;37–42.

[32] Berkhof M, van Rijssen HJ, Schellart AJM, van der Anema JR (2011). Effective training strategies for teaching communication skills to physicians: an overview of systematic reviews. Patient Educ Couns.

[33] Lane C, Rollnick S (2007). The use of simulated patients and role-play in communication skills training: a review of the literature to August 2005. Patient Educ Couns Patient ed.

