# Supplementary material for: Online vs. face-to-face interactive communication education using video materials among healthcare college students: a pilot non-randomized controlled study
Source: BMC Med Educ. 2024 Jul 10;24:746. doi: 10.1186/s12909-024-05742-2 (PMC11238445; doi:10.1186/s12909-024-05742-2)
Supplement: Supplementary file 1 — Supplementary Material 1 [file 12909_2024_5742_MOESM1_ESM.docx]

Appendix1. E-learning homework of listening skills


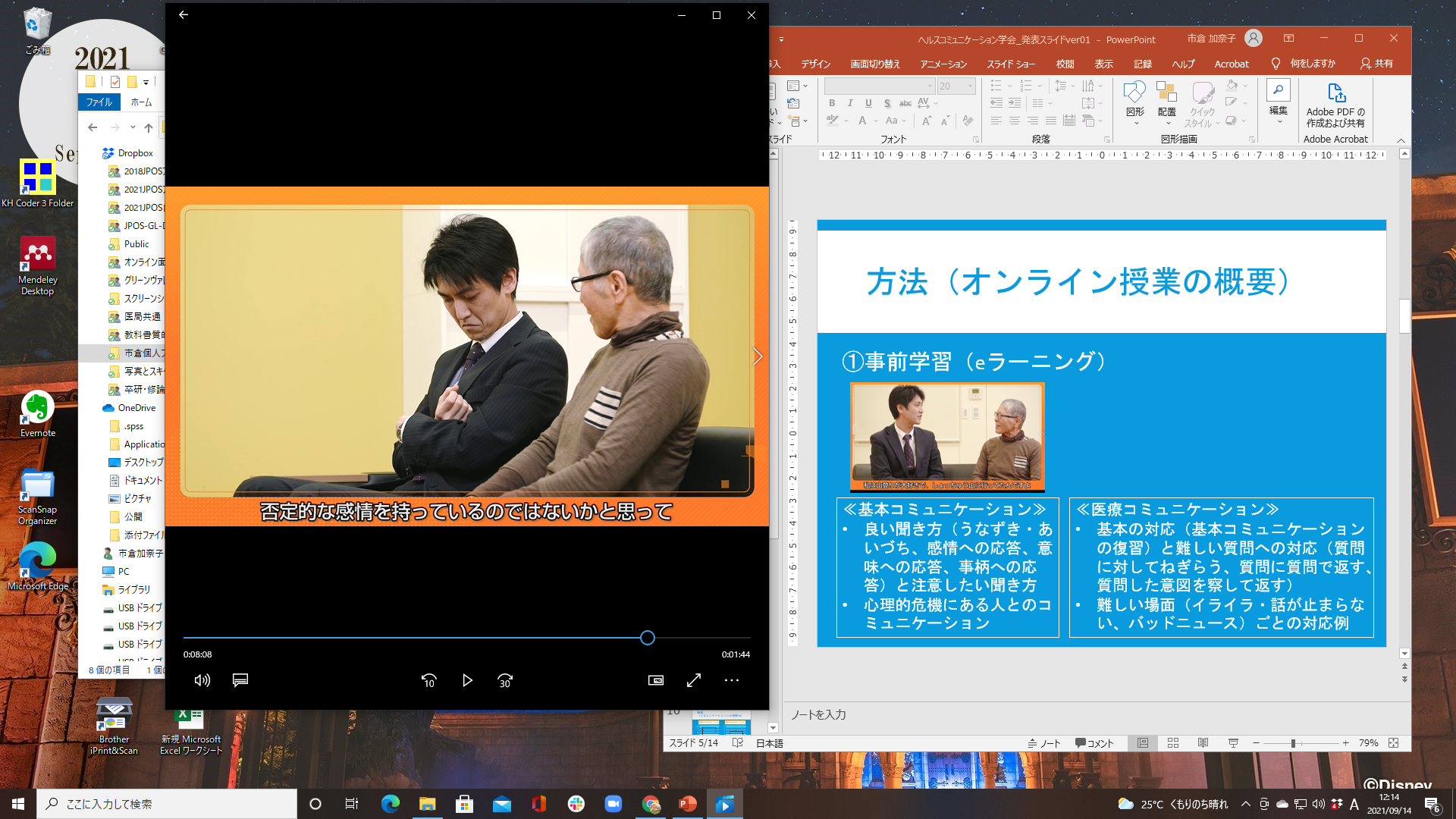

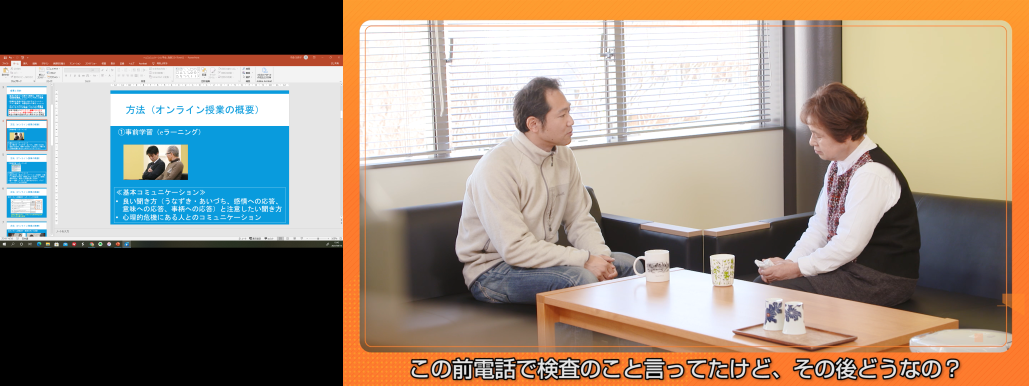


Not making eye contact with the other person

Strong tone of voice and blame

（Example of poor listening skills)

Appendix2. Confirmation quiz of listening skills

In the following conversation, choose the reflection technique corresponding to the phrase from A to D and answer with a symbol.

A: Nodding/back-channeling

B: Reflection of feeling

C: Reflection of meaning

D: Reflection of content

H (Health care provider): Hello. How have you been since we met the other day?

P (Patient): Hello. I have not been feeling well since the day before yesterday.

H: You have not been feeling well since the day before yesterday. (1. )

P: I failed the employment exam.

H: So it was. Your employment did not go well? (2. )

P: Yes. I was very disappointed because I thought I would be able to pass the exam thit time from the test and interview.

H: I see, that is disappointing. (3. )

P: I can't think of any reason for the rejection. Then, I looked back at the rejection notices and it becomes painful.

H: So you feel hard by looking back at the notice. (4. )

P: Exactly. Perhaps it is a psychological effect, but I have a stomach ache.

H: So you have a stomach ache. (5. )

P: I hope I can get a job at the next place I’m applying to.

H: Yes, it really is. (6. )

Appendix3. Practical exercise of listening skills


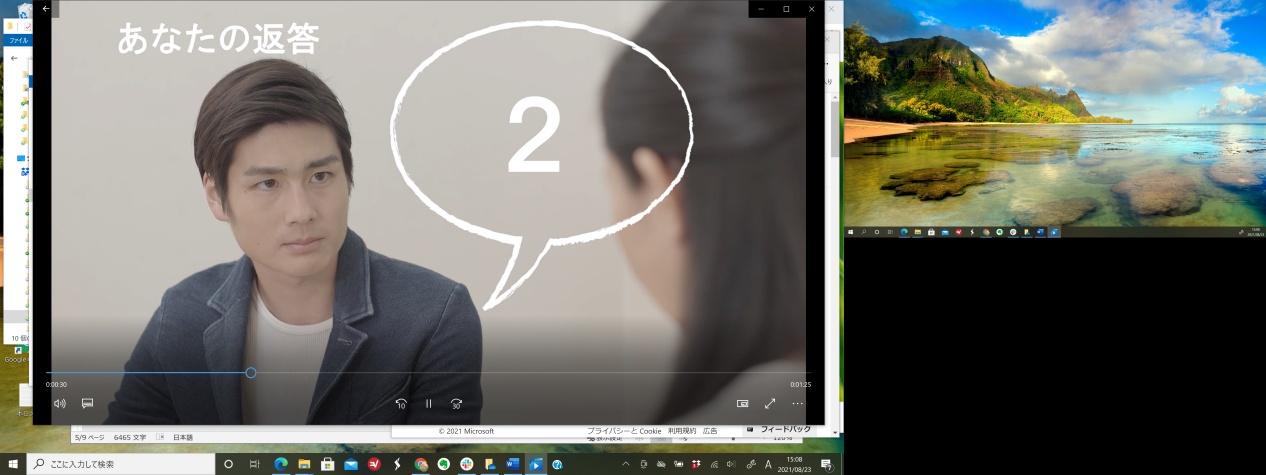

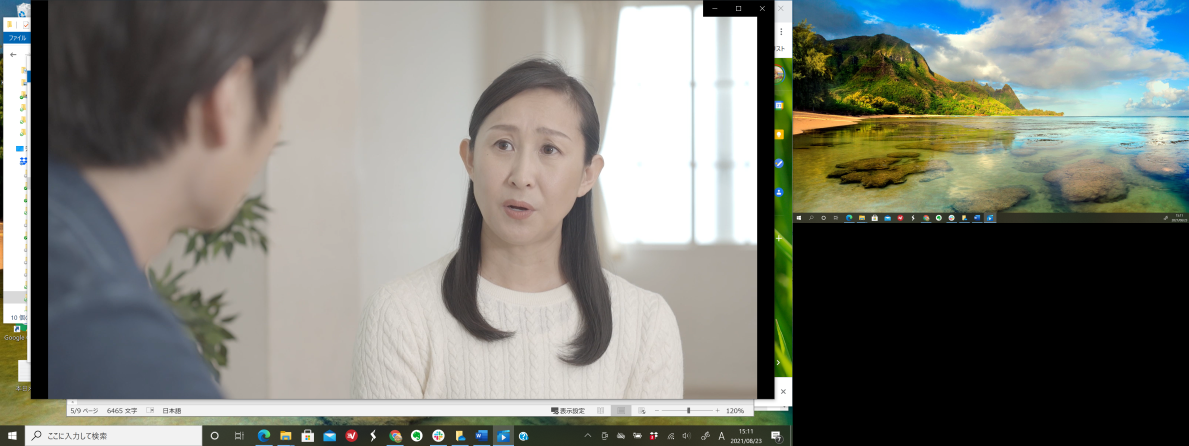


I’ve been so tired lately.

Your response

（Conversation video without voice of the health care provider)
